# Derangements of immunological proteins in HIV-associated diffuse large B-cell lymphoma: the frequency and prognostic impact

**DOI:** 10.3389/fcimb.2024.1340096

**Published:** 2024-04-03

**Authors:** Jenifer Vaughan, Moosa Patel, Melinda Suchard, Maemu Gededzha, Heena Ranchod, Wayne Howard, Tracy Snyman, Tracey Wiggill

**Affiliations:** ^1^ Department of Molecular Medicine and Haematology, Faculty of Health Sciences, University of the Witwatersrand, Johannesburg, South Africa; ^2^ National Health Laboratory Services, Johannesburg, South Africa; ^3^ Department of Medicine, Faculty of Health Sciences, University of the Witwatersrand, Johannesburg, South Africa; ^4^ Clinical Haematology Unit, Chris Hani Baragwanath Academic Hospital, Johannesburg, South Africa; ^5^ Department of Chemical Pathology, Faculty of Health Sciences, University of the Witwatersrand, Johannesburg, South Africa; ^6^ Department of Immunology, University of the Witwatersrand, Johannesburg, South Africa; ^7^ National Institute for Communicable Diseases, Centre for Vaccines and Immunology, Johannesburg, South Africa

**Keywords:** HIV-associated DLBCL, cytokines, IL-6, IL-10, TGFβ, ferritin, CRP, Indoleamine 2,3-dioxygenase

## Abstract

**Introduction:**

Diffuse large B-cell lymphoma (DLBCL) is an aggressive malignancy of B-cells frequently encountered among people living with HIV. Immunological abnormalities are common in immunocompetent individuals with DLBCL, and are often associated with poorer outcomes. Currently, data on derangements of immunological proteins, such as cytokines and acute phase reactants, and their impact on outcomes in HIV-associated DLBCL (HIV-DLBCL) is lacking. This study assessed the levels and prognostic relevance of interleukin (IL)-6, IL-10 and Transforming Growth Factor Beta (TGFβ), the acute phase proteins C-reactive protein (CRP) and ferritin; serum free light chains (SFLC) (elevation of which reflects a prolonged pro-inflammatory state); and the activity of the immunosuppressive enzyme Indoleamine 2,3-dioxygenase (IDO)in South African patients with DLBCL.

**Methods:**

Seventy-six patients with incident DLBCL were enrolled, and peripheral blood IL-6, IL-10, TGFβ, SFLC and IDO-activity measured in selected patients. Additional clinical and laboratory findings (including ferritin and CRP) were recorded from the hospital records.

**Results:**

Sixty-one (80.3%) of the included patients were people living with HIV (median CD4-count = 148 cells/ul), and survival rates were poor (12-month survival rate 30.0%). The majority of the immunological proteins, except for TGFβ and ferritin, were significantly higher among the people living with HIV. Elevation of IL-6, SFLC and IDO-activity were not associated with survival in HIV-DLBCL, while raised IL-10, CRP, ferritin and TGFβ were. On multivariate analysis, immunological proteins associated with survival independently from the International Prognostic Index (IPI) included TGFβ, ferritin and IL-10.

**Conclusion:**

Derangements of immunological proteins are common in HIV-DLBCL, and have a differential association with survival compared to that reported elsewhere. Elevation of TGFβ, IL-10 and ferritin were associated with survival independently from the IPI. In view of the poor survival rates in this cohort, investigation of the directed targeting of these cytokines would be of interest in our setting.

## 1 Introduction

Diffuse large B-cell lymphoma (DLBCL) is an aggressive malignancy of B-cells, which constitutes the most common subtype of non-Hodgkin lymphoma worldwide. Immune dysfunction contributes to the pathogenesis of DLBCL, and it is consequently seen with higher frequency among patients with immunodeficiency, including people living with HIV infection. Immunological abnormalities have also been described in DLBCL due to autocrine cytokine production by the tumour cells (Lam et al., 2008; Nie et al., 2019; Manfroi et al., 2021), paracrine cytokine production by surrounding immune and mesenchymal cells (Cha et al., 2017; Zhong et al., 2019), and T-cell exhaustion. The latter occurs due to chronic antigenic stimulation (Pauken and Wherry, 2015) and/or the expression of co-inhibitory receptors, such programmed cell death ligand 1 (PD-L1) on the tumour cells (Xing et al., 2016). The immune constituents of the tumour microenvironment (TME) are known to influence tumour behaviour, with a dominantly anti-inflammatory TME thought to promote immune evasion and tumour growth. For instance, tumour enrichment with anti-inflammatory (M2) macrophages has been reported to be associated with inferior outcomes (Nam et al., 2014; Marchesi et al., 2015). Peripheral blood levels of some cytokines have been shown to correlate with those measured within the tumour (Voorzanger et al., 1996), thus possibly accounting for the negative impact on outcomes associated with high peripheral blood levels of the anti-inflammatory cytokine interleukin (IL)-10 (Lech-Maranda et al., 2006; Načinović-Duletić et al., 2008; Gupta et al., 2012), as well as the regulatory T-cell (Treg)-inducing enzyme Indoleamine 2,3-dioxygenase (IDO) (Yoshikawa et al., 2010; Chen et al., 2020). Somewhat counter-intuitively, high peripheral blood levels of pro-inflammatory cytokines (such as IL-6) are also associated with inferior outcomes in DLBCL (Načinović-Duletić et al., 2008; Giachelia et al., 2012). This may be due in part to the homeostatic anti-inflammatory response which follows a pro-inflammatory stimulus, serving to prevent the tissue damage which could ensue from an unrestrained immune response. In people living with HIV, chronic or recurrent infections would be anticipated to alter the immune milieu, which may contribute to the poorer outcomes sometimes seen in HIV-associated DLBCL (HIV-DLBCL). To date, there has been sparse research with respect to immunological derangements in the setting of HIV-DLBCL. We have previously shown differential effects of immune-cell abnormalities in a South African cohort of patients with DLBCL and a high HIV-prevalence as compared to that reported in immunocompetent patients. For instance, while high numbers of Tregs, lymphopenia, low CD4 counts and neutrophilia were associated with inferior outcomes, high monocyte counts were not (Vaughan et al., 2023). In this follow-up study on the same cohort, we aimed to assess the levels and prognostic relevance of immunological proteins previously shown to have a prognostic impact in DLBCL among dominantly immunocompetent patients. These include: the pro-inflammatory cytokine interleukin IL-6; the acute phase proteins C-reactive protein (CRP) and ferritin; serum free light chains (SFLC), elevation of which reflects a prolonged pro-inflammatory state; the anti-inflammatory cytokines IL-10 and Transforming Growth Factor Beta (TGFβ); and the plasma kynurenine/tryptophan ratio (KTR), an indirect measure of the activity of the immunosuppressive enzyme IDO.

## 2 Methods

Seventy-six adult patients with newly diagnosed DLBCL at Chris Hani Baragwanath Academic Hospital (CHBAH) in Johannesburg were enrolled between November 2019 and May 2022 as reported previously (Vaughan et al., 2023). Where possible, IL-6, IL-10, TGFβ, SFLC and KTR levels were measured on a peripheral blood sample prior to the commencement of chemotherapy. The number of tests performed and the reasons for test omission are summarized in **Supplementary Figure 1**. IL-6, IL-10 and TGFβ were measured in replicate wells using MILLIPLEX^®^ MAP Human Cytokine/Chemokine magnetic bead panel assays (Merck KGaA, Darmstadt, Germany) on a Luminex^®^ 200™ (Luminex Corporation, Austin, USA). SFLC levels were measured immunoturbidometrically on a Beckmann Coulter IMMAGE analyser (Beckman Coulter, Inc, Brea, California, USA) using the FREELITE assay (The Binding Site Ltd, Birmingham, United Kingdom). The KTR was measured in replicate wells using Kynurenine and Tryptophan ELISA kits (ImmuSmol, France), both levels converted to nmol/L and the KTR ratio calculated. ferritin and CRP were measured using a Cobas 8000 e602 module and a Cobas 8000 c702/c502 module, respectively (Roche Products, Basel, Switzerland). Treg numbers and expression of CD39 and Helios were measured in a subset of patients using a DuraClone IM Treg antibody panel (Beckman Coulter Inc, Brea, USA) (as described previously) (Vaughan et al., 2023). Additional relevant information was retrieved from the laboratory information system (TrakCare, InterSystems, Cambridge, Massachusetts, United States) and the patient hospital records. This included details of the clinical presentation, stage and treatment, Eastern Cooperative Oncology Group (ECOG) performance status (PS), the international prognostic index (IPI) score, HIV-status and associated information [Anti-retroviral therapy (ART) exposure, the CD4-count and HIV viral load (HIVVL)], histology and bone marrow (BM) findings, full blood count and differential white cell counts, biochemistry results (including lactate dehydrogenase (LDH), CRP, ferritin and immunoglobulins), and information about the clinical course and outcomes. Some of the data were unavailable in some patients owing to lost or incomplete hospital records/work-up. The cell of origin (COO) (Germinal Centre (GC) versus non-GC) was determined by means of the Hans algorithm (Hans et al., 2004). The study was approved by the Human Research Ethics Committee (HREC) of the University of the Witwatersrand (reference number M190709). Informed consent was obtained from all participants from whom peripheral blood was collected solely for the purposes of the study, as well as from all patients on whom testing was performed on residual samples, provided they had not demised in the interim.

### 2.1 Statistical analysis

The Fishers exact test (categorical) and Mann-Whitney U-test (continuous) were used to compare clinical and laboratory data according to HIV-status, and Spearman’s correlation was used to assess for correlation between variables of interest. Cut-off values for the IL-6, IL-10, TGFβ, KTR, Kynurenine, Tryptophan, Ferritin and CRP levels were determined using Receiver Operator Characteristics (ROC) curve analysis. The cut-off values were selected based on a high likelihood ratio (LR) with the highest possible sensitivity and specificity. The one-year overall survival (OS) rate reflects the proportion of patients alive one year after diagnosis. Patients who were alive but had been followed up for less than a year were excluded from this analysis, as were clinically stable patients who were lost to follow-up before commencing treatment. Kaplan-Meier survival estimates were used to perform univariate survival analysis, and log rank tests were used to compare median survival times in the entire cohort and in the people living with HIV. This was done to more closely assess determinants of survival in HIV-DLBCL. The limited number of HIV-negative patients included in the study precluded similar sub-analysis in this group. Multivariate survival analysis was performed using Cox proportional-hazards models, which included predictor variables which showed a statistically significant association with survival on Kaplan-Meier analysis. As sample numbers were too low to include all the significant variables in one multivariate model, multiple Cox proportional-hazards analyses were performed, including the variable of interest and the IPI, with or without the CD4 count. Schoenfield analysis of proportionality was used to verify that the assumptions of proportionality were met. Statistical analysis was performed using Prism software, version 5 (GraphPad Software, San Diego, California, United States), at https://statpages.info/prophaz.html (Cox proportional hazard regression analysis) and at https://acetabulum.dk/cgi-bin/cox (proportionality testing). Statistical significance was accepted at a two-sided p-value of 0.05.

## 3 Results

Among the 76 patients included, HIV-status was documented in 75 (98.7%), with an HIV-prevalence of 81.3% (61/75). The median age of the cohort was 42 years, although the HIV-negative patients were significantly older (**Table 1**). A history of ART-exposure was available in 56/61 patients; 46 of these patients (82.1%) were on ART at the time of referral. The median CD4 count was 148 cells/ul, and 67.2% (41/61) patients had a count of <200 cells/ul at presentation. The majority of patients, both HIV-negative and people living with HIV, had stage IV disease. Data regarding treatment were available in 68 patients, of whom 58 (85.3%) received chemotherapy. The majority (46) received a cyclophosphamide, doxorubicin, vincristine, and prednisone (CHOP) based chemotherapy protocol without rituximab upfront; firstline rituximab was added to CHOP in 8 HIV negative patients (**Table 1**). Intrathecal chemotherapy was administered to 15 patients on the basis of documented central nervous system (CNS) involvement (one patient) or due to lymphomatous involvement of the head or neck. Survival data was available in 73 patients; at a median follow-up period of 29.5 months (IQR 17-30.8 months), the median survival time from diagnosis was 4 months, with a 12-month survival rate of 31.5%. This poor survival was largely attributable to a very high early death rate, with 19 patients (26.0% of all those with survival data available) dying ≤1 month after diagnosis. Among these patients 9 (56.3% of those with available treatment data) demised before chemotherapy could be commenced. The most common cause of death among the patients who died early was suspected or confirmed sepsis [8/19 (42.1%)], followed by renal failure [3/19 (15.8%)]. One patient (5.3%) succumbed to tumour lysis syndrome, while the cause of death was unknown in 7/19 (36.8%) of these patients. LDH levels were significantly higher among the patients who died early as compared to those who survived for more than one month (1168 U/L vs 585 U/L; p= 0.002), the CD4 counts were significantly lower (97 versus 180.5 cells/µL; p=0.019), the proportion of patients with a poor ECOG performance status was significantly higher (91.7% versus 37.0%; p=0.0008) and the proportion of patients on ART was significantly lower (57.1% versus 90.5%; p=0.01). Relevant demographic and clinical data is summarised in **Table 1**.

**Table 1:** Demographic and clinical data.

Parameter	Result	p-value*
Age, median (IQR) HIV+ HIV-	42 (35-54) 41 (33.5-49) 60 (34.8-63.3)	**0.02**
Male : Female HIV+ HIV-	1.3:1 1.2:1 1.8:1	0.56
HIV seropositive, n/N (%)	61/75 (81.3)	/
CD4 (cells/ul), median (IQR) (n=70) Φ HIV+ (N=61) HIV- (N=8)	148 (74-312) 143 (70-282) 251 (149-767)	0.057
HIVVL (cps/ml), median (IQR) (n=56)	189 (20-118752)	/
Virological suppression^ψ^, n/N (%)	21/56 (37.5%)	/
ART exposure, n/N (%)	46/56 (82.1%)	/
Stage IV disease, n/N (%) HIV+ HIV-	48/62 (77.4) 40/49 (81.6) 8/13 (61.5)	0.63
IPI 4-5, n/N (%) HIV+ HIV-	17/58 (29.3) 12/45 (26.7) 5/13 (38.5)	0.49
Performance status ≥2, n/N(%) HIV+ HIV-	28/58 (53.8) 21/45 (46.7) 7/13 (53.8)	0.89
Bulky disease HIV+ HIV-	25/55 (45.5) 18/42 (42.9) 7/13 (53.8)	0.54
Extranodal diseaseÞ, n/N (%)Φ HIV+ HIV-	54/68 (79.4) 40/53 (75.5) 13/14 (92.9)	0.27
Bone marrow involvement, n/N (%) HIV+ HIV-	13/65 (20) 9/51 (17.6) 4/14 (28.6)	0.45
Cell of origin GC^α^, n/N (%)Φ HIV+ HIV-	39/52 (75) 32/41 (78.1) 7/10 (70)	0.68
Ki-67 (%), median (IQR) (n=71) Φ HIV+ (n=56) HIV- (n=14)	90 (80-95) 90 (80-95) 90 (89-95)	0.99
LDH (U/L), median (IQR) (n=70) HIV+ (n=56) HIV- (n=14)	719 (417-1289) 725 (448-1295) 591 (301-1293)	0.29
Β_2_microglobulin (mg/L), median (IQR) (n=57) HIV+ (n=46) HIV- (n=11)	4.7 (3.15-7.15) 4.2 (2.7-7.8) 4.8 (3.3-7.0)	0.64
Index chemotherapy protocol, n/N (%) CHOP-based R-CHOP based Other combination chemotherapy regimens	45/57(78.9) 8/57 (14.0) 4/57 (7.0)	/

HIVVL, HIV viral load; ART, antiretroviral therapy; IPI, International prognostic index; CHOP, cyclophosphamide, doxorubicin, vincristine, and prednisone; R-CHOP:, rituximab plus CHOP; GC, germinal center subtype. *p-values derived from analysis of HIV+ vs HIV-. Φ, One patient was of unknown HIV-status. ψ, HIVVL<100cps/ml, α, according to the Hans algorithm. The balance of the cases were of non-GC origin. Þ Extranodal disease includes cases with bone marrow, liver and spleen involvement. The bold p-values are statistically significant.

### 3.1 IL-6, IL-10 and TGFβ1 results

IL-6 levels were universally elevated in the patients included in this cohort (median 381.6 pg/ml (IQR 207.2-1452)) as compared to normal levels reported in the literature (4.63-5.74 pg/mL) (Said et al., 2021), and were significantly higher among the people living with HIV compared to the HIV-negative patients (**Table 2**). There was a significant positive correlation between the IL-6 level and the CRP (r_s_ = 0.40, p = 0.03) and a trend to an inverse correlation between IL-6 levels and bulky disease which did not meet statistical significance (r_s_ -0.33, p=0.08). No significant correlations with the CD4 count, HIVVL, disease stage, LDH, haemoglobin level or the COO were present. On survival analysis, there was no significant association between the IL-6 levels and survival (**Table 3**).

**Table 2:** Immunological protein data.

Parameter	Reference Range	Result	p-value*
IL-6 (pg/mL), median (IQR) n=(36) Φ HIV+ (n=26) HIV- (n=9)	4.63-5.74 pg/mL Said et al., 2021	381.6 (207.2-1452) 536.6 (266-2171) 290.3 (113.3-411)	**0.048**
IL-10 (pg/mL), median (IQR) n=(36) Φ HIV+ (n=26) HIV- (n=9)	<20 pg/ml (Sarris et al., 1999; Fayad et al., 2001; Nemunaitis et al., 2001; Načinović-Duletić et al., 2008; Gupta et al., 2012)	184.5 (66.9-559.5) 234.5 (114.3-857.2) 71 (29-393.6)	0.067
TGFβ1 (pg/mL), median (IQR) n=(36) Φ HIV+ (n=26) HIV- (n=9)	Unknown	1083 (21.6-4653) 1879 (21.6-4696) 21.6 (9.8-4905)	0.31
C-reactive protein (mg/L), median (IQR) (n=67) Φ HIV+ (n=54) HIV- (n=12)	<10 mg/L	93 (45-160) 109 (52-200) 68 (20-91)	**0.039**
Ferritin (µg/L), median (IQR) (n=61) Φ HIV+ (n=50) HIV- (n=10)	30-150 µg/L	405 (194-988) 401 (207-976) 423 (110-834)	0.49
"Serum free Kappa (mg/L), median (IQR) (n=63) HIV+ (n=52) HIV- (n=11)	3.3-19.4 mg/L	32.1 (16.4-58.0) 34.0 (20.3-63.0) 15.2 (10.5-32.7)	**0.037**
Serum free Lambda (mg/L), median (IQR) (n=63) HIV+ (n=52) HIV- (n=11)	5.7-26.3 mg/L	42.6 (21.8-71.8) 48.5 (24.7-79.2) 16.2 (10.6-27.9)	**0.037**
Kynurenine (nmol/L), median (IQR) (n=61) Φ HIV+ (n=47) HIV- (n=13)	Unknown	3389 (2474-4589) 3551 (2797-4575) 2783 (2198-3309)	**0.034**
Tryptophan (nmol/L), median (IQR) (n=61) Φ HIV+ (n=47) HIV- (n=13)	Unknown	30137 (16675-45863) 28837 (15618-46544) 33714 (25513-43031)	0.40
K/T ratio (KTR) (n=61) Φ HIV+ (n=47) HIV- (n=13)	<0.1	0.10 (0.07-0.20) 0.12 (0.08-0.21) 0.08 (0.06-0.09)	**0.045**

Φ, One patient was of unknown HIV-status. The bold p-values are statistically significant.

**Table 3:** Survival analysis.

	Median survival entire cohort (months)	Median survival people living with HIV (months)
**IPI** ≥4 <4 Hazard ratio (CI) p-value	2.75 13 5.08 (2.09-12.40) **0.0003** (N=54)	1.75 9 5.40 (1.87-15.60) **0.0018** (N=41)
**CD4** <150 cells/ul ≥150 cells/ul Hazard ratio (CI) p-value	3 Not reached 2.72 (1.43-5.18) **0.002** (N=67)	2.75 14 2.36 (1.22-4.54) **0.01** (N=59)
**IL-6** ≥633.3 pg/ml <633.3 pg/ml Hazard ratio (CI) p-value	4 4 0.83 (0.34 – 2.01) 0.68 (N=35)	4 2.75 0.69 (0.26-1.84) 0.46 (N=26)
**IL-10** ≥129.3 pg/ml <129.3 Hazard ratio (CI) p-value	3 Not reached 5.52 (2.28-13.37) **0.0002** (N=35)	2.75 Not reached 3.99 (1.43-11.14) **0.008** (N=26)
**TGFβ1** ≥1083 pg/ml <1083 Hazard ratio (CI) p-value	2.75 18 2.65 (1.08-6.32) **0.033** (N=35)	1.75 14 3.11 (1.12-8.59) **0.029** (N=26)
**CRP** ≥75.5 mg/L <75.5 Hazard ratio (CI) p-value	2.75 14 2.1 (1.16-3.83) **0.01** (N=67)	1.88 13 2.08 (1.07-4.03) **0.03** (N=52)
**Ferritin** ≥324 µg/L <324 Hazard ratio (CI) p-value	3 Not reached 3.29 (1.68-6.47) **0.0005** (N=61)	2.75 Not reached 2.86 (1.38-5.94) **0.005** (N=50)
**SFLC** Any abnormality Normal Hazard ratio (CI) p-value	4 9.5 1.41 (0.69-2.89) 0.34 (N=61)	3 5 1.23 (0.53-2.86) 0.63 (N=50)
**SFLC** Both LCs increased No or one LC increased Hazard ratio (CI) p-value	3 7 1.37 (0.73-2.59) 0.33 (N=61)	3 9 1.52 (0.75-3.08) 0.24 (N=50)
**SFLC** Abnormal SFLC ratio Normal SFLC ratio Hazard ratio (CI) p-value	9 3.75 0.97 (0.40-2.34) 0.95 (N=61)	27.5 3 0.65 (0.24-1.78) 0.40 (N=50)
**Kynurenine** ≥3438 nmol/L <3438 Hazard ratio (CI) p-value	4.3 4 1.02 (0.54-1.93) 0.95 (N=60)	4 3.75 0.87 (0.43-1.77) 0.70 (N=46)
**Tryptophan** <33504 nmol/L ≥33504 Hazard ratio (CI) p-value	4 14 1.58 (0.83-3.0) 0.16 (N=60)	3 9.4 1.61 (0.79-3.28) 0.19 (N=46)
**KTR** ≥0.092 <0.092 Hazard ratio (CI) p-value	3 5 1.63 (0.86-3.09) 0.14 (N=60)	2.75 9.5 1.75 (0.86-3.57) 0.13 (N=46)

IPI, International prognostic index; SFLC, serum free light chains; KTR, K:T ratio. The bold p-values are statistically significant.

**Figure 1:**
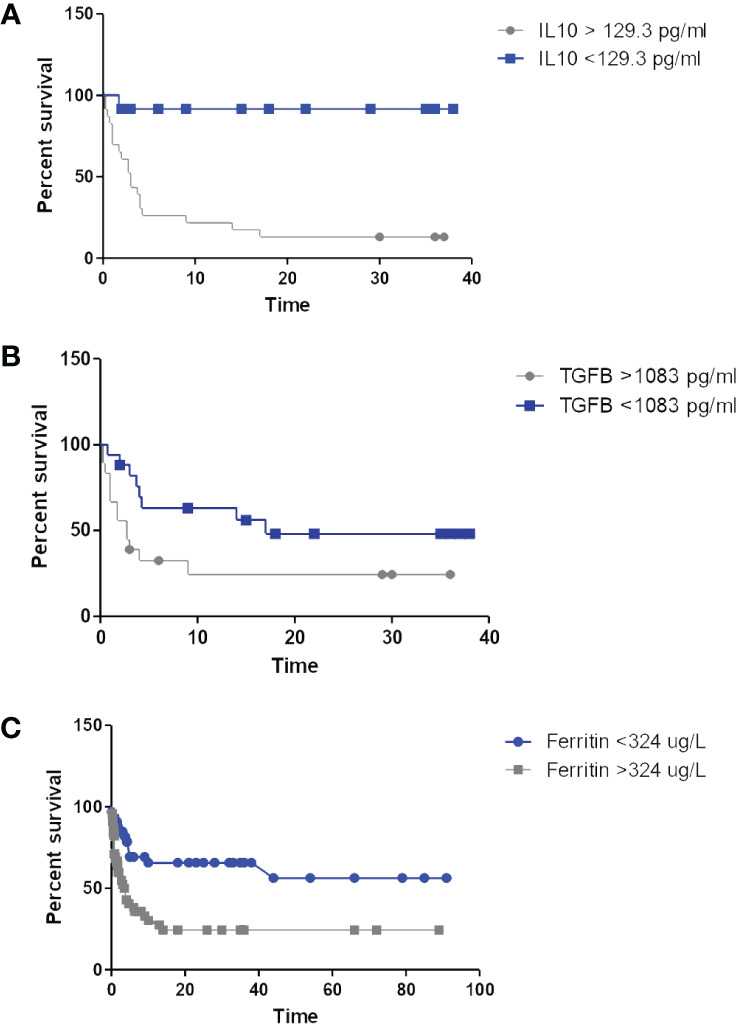
Kaplan-Meier curves depicting survival according to IL-10 **(A)**, TGFβ **(B)** and ferritin levels **(C)**.

IL-10 levels were elevated in all patients included in this study as compared to the normal levels reported in the literature (<20 pg/ml) (Sarris et al., 1999; Fayad et al., 2001; Nemunaitis et al., 2001; Načinović-Duletić et al., 2008; Gupta et al., 2012), and were marginally higher among the people living with HIV (**Table 2**). Significant correlations were present between the IL-10 levels and the Albumin (r_s_=-0.62, p<0.001), Haemoglobin (r_s_=-0.42, p=0.011), monocyte counts (r_s_ = 0.32, p= 0.021) and IL-6 levels (r_s_=0.4, p=0.016), with no correlations evident between the IL-10 levels and the COO, the Treg count, the LDH, the IPI, β2-microglobulin levels, bulky disease or the CD4 count. Marginally significant correlations were seen between the IL-10 level and the CRP (r_s_=0.38, p=0.08) and disease stage (r_s_=0.35, p=0.067). Survival times were significantly shorter in patients with IL-10 levels above 129.3 pg/ml (**Table 3**; **Figure 1A**).

Normal serum levels of TGFβ1 are not well established, however, median TGFβ1 levels in this cohort showed no significant difference according to HIV-status (**Table 2**), and were significantly correlated with the neutrophil (r_s_=0.4, p=0.015) and monocyte (r_s_=0.5, p= 0.002) counts. In addition, TGFβ levels were marginally correlated with the performance status (r_s_ = 0.377, p=0.07) and bulky disease (r_s_ = 0.308, p=0.097), but showed no relationship to age, disease stage, IPI, HIV-related parameters (including CD4 counts), CRP, ferritin, IL-6 or IL-10 levels. No correlation was seen between the TGFβ levels and the Treg count, but there were significant inverse correlations between TGFβ and Treg expression of CD39 (r_s_ = -0.6, p=0.003) and), and Helios (r_s_ = -0.53, p=0.011), both of which are associated with a more potent immunosuppressive regulatory T-cell phenotype (Elkord et al., 2016; Gu et al., 2017). On univariate survival analysis, levels >1083 pg/ml were found to be significantly associated with inferior survival among the people living with HIV (**Table 3**; **Figure 1B**).

### 3.2 CRP and ferritin results

Both median CRP and ferritin levels were elevated in this cohort of patients, with only 5/67 (7.5%) and 12/61 (19.7%) having CRP and ferritin levels within the normal range, respectively. CRP levels were significantly higher among the people living with HIV, while ferritin levels were not. As expected, ferritin and CRP levels correlated significantly with each other (r_s_ 0.37, p =0.015), and both also showed significant correlations with disease stage, LDH levels, β2 microglobulin levels, albumin, haemoglobin and the CD4 count (**Table 4**). CRP (but not ferritin) showed a significant correlation with the IL-6 levels, while ferritin (but not CRP) was significantly correlated with IL-10 levels. In addition, ferritin (but not CRP) was significantly correlated with the performance status and IPI score.

**Table 4:** Spearman’s correlation results for ferritin and CRP against other variables of interest.

Parameter	Ferritin Correlation co-efficient (r_s_) (p-value)	CRP Correlation co-efficient (r_s_) (p-value)
Age	0.24 (0.054)	-0.06 (0.64)
Performance status	0.34 (**0.031**)	0.24 (0.121)
IPI	0.35 (**0.02**)	0.17 (0.26)
Stage	0.34 (**0.015**)	0.37 (**0.006**)
LDH	0.36 (**0.007**)	0.62 **(<0.001**)
Albumin	-0.34 (**0.008**)	-0.35 (**0.004**)
β2-microglobulin	0.50 (**<0.001**)	0.37 (**0.008**)
Hb	-0.29 (**0.026**)	-0.33 (**0.007**)
CD4	-0.28 (**0.034**)	-0.33 (**0.01**)
IL-6	-0.006 (0.98)	0.4 (**0.028**)
IL-10	0.37 (**0.047**)	0.223 (0.24)
CRP	0.33 (**0.015**)	/

The bold p-values are statistically significant.

On univariate survival analysis, elevated levels of both ferritin (**Figure 1C**) and CRP were significantly associated with shorter survival in the people living with HIV (**Table 3**).

### 3.3 SFLC results

The median levels of both serum free Kappa and Lambda light chains were well above the normal range in this cohort of patients. This was owing to significantly higher free light chain (FLC) levels among the people living with HIV, while FLC levels were within the normal range in the HIV negative group (**Table 2**). SFLC results were abnormal in 49 of the 64 patients tested (76.6%). Among these, 10 (20.4%) had elevation of one free light chain level (either Kappa or Lambda), and 39 (79.6%) had elevated levels of both light chains. An abnormal SFLC ratio was present in 9 (14.1%) patients, of whom 4 had elevation of only one light chain, and 5 had elevation of both. The frequency of normal light chain levels was marginally higher in the HIV-negative individuals, and the presence of elevation of both light chains was significantly higher among the people living with HIV (**Table 5**). The frequency of elevation of one light chain and an abnormal light chain ratio did not differ significantly according to HIV-status. Abnormalities of the SFLC ratio were correlated with age (r_s_ 0.282, p=0.024) and the IPI score (r_s_ 0.325, p= 0.033), while elevation of both light chains was correlated with β2-microglobulin levels (r_s_ = 0.424, p = 0.003), the CRP (r_s_= 0.283, p = 0.033), IgG levels (r_s_= 0.59, p = 0.002) and CD4 count (r_s_= -0.273, p=0.035), but not serum creatinine, age, IL-6 or IL-10 levels. On univariate survival analysis, abnormalities of the SFLC were not significantly associated with survival in the people living with HIV (**Table 3**).

**Table 5:** SFLC-related data.

	All patients (n=64) Φ	Patients living with HIV (n=52)	HIV-negative patients (n=11)	p-value
Normal SFLC levels [n(%)]	15 (23.4)	9 (17.3)	5 (45.5)	0.056
Elevation of one free light chain level	10 (15.6)	7 (17.3)	3 (27.3)	0.36
Elevation of both free light chain levels	39 (60.9)	36 (69.2)	3 (27.3)	**0.015**
Abnormal SFLC ratio	9 (14.0)	6 (11.5)	3 (27.3)	0.34

Φ: One patient was of unknown HIV-status. The bold p-values are statistically significant.

### 3.4 Activity of the immunosuppressive enzyme indoleamine 2,3-dioxygenase

The KTR was above the reported upper limit of the normal range (0.0-0.1) (Adu-Gyamfi et al., 2021) in 47.5% (29/61) of the patients in this cohort, and was significantly higher among the people living with HIV (**Table 2**). Significant correlations were present between the KTR and advanced disease stage (r_s_=0.41, p =0.03), β2-microglobulin levels (r_s_ = 0.33, p=0.025) and ferritin (r_s_=0.3, p=0.006), while no significant relationships were seen between the KTR and HIV-related parameters (including the CD4 count), any of the measured cytokine levels or the Treg count. Median Kynurenine levels were considerably higher (3389 nmol/L) than those reported in a previously study among Chinese patients with DLBCL (1575 nmol/L) (Yoshikawa et al., 2010), and were significantly higher among the people living with HIV. Kynurenine levels were correlated with the Treg count (r_s_ = 0.53, p=0.003) and showed a marginal correlation with the TGFβ levels (r_s_ = 0.279, p =0.099), but not with the disease stage, performance status, IPI or HIV-related parameters. On univariate survival analysis, neither the KTR nor serum Kynurenine levels showed any significant association with survival in the people living with HIV.

### 3.5 Multivariate survival analysis

As variables significantly associated with survival on univariate analysis, ferritin >324 µg/L, CRP>75.5 mg/L, IL-10 >129.3 pg/ml and TGFβ>1083 pg/ml were all included in separate Cox proportional-hazards models with the IPI (since the sample size was too small to include all of these variables in a single analysis). For CRP and ferritin, a CD4 count <150 cells/ul was also included in the model, as the CD4 count had a strong association with survival, and showed a significant correlation to both of these variables. These analyses showed the ferritin (**Table 6**), TGFβ (**Table 7**) and IL-10 (**Table 8**) to be significantly associated with survival independently from the IPI and the CD4 count (in the case of the ferritin level), while the CRP was not (**Table 9**).

**Table 6:** Cox proportional-hazards regression analysis for independent associations between the survival time and the ferritin level and the IPI.

	Coefficient	95% Confidence interval	p-value	Hazard ratio	95% Confidence interval
Ferritin ≥324 µg/L	1.10	0.17 to 2.03	**0.02**	3.0	1.19 to 7.58
IPI ≥4	0.63	-0.21 to 1.47	0.14	1.88	0.81 to 4.34
CD4 <150 cells/ul	0.32	-0.55 to 1.18	0.47	1.38	0.58 to 3.27

The bold p-values are statistically significant.

**Table 7:** Cox proportional-hazards regression analysis for independent associations between the survival time and the TGFβ level and the IPI.

	Coefficient	95% Confidence interval	p-value	Hazard ratio	95% Confidence interval
TGFB ≥1083 pg/ml	1.26	0.18 to 2.34	**0.02**	3.54	1.20 to 10.42
IPI ≥4	1.01	-0.03 to 2.05	0.06	2.75	0.97 to 7.79

The bold p-values are statistically significant.

**Table 8:** Cox proportional-hazards regression analysis for independent associations between the survival time and the IL-10 level and the IPI.

	Coefficient	95% Confidence interval	p-value	Hazard ratio	95% Confidence interval
IL-10 ≥129.3 pg/ml	2.49	0.44 to 4.54	**0.02**	12.0	1.55 to 93.74
IPI ≥4	0.26	-0.76 to 1.28	0.61	1.30	0.47 to 3.60

The bold p-values are statistically significant.

**Table 9:** Cox proportional-hazards regression analysis for independent associations between the survival time and the CRP level, CD4 count and the IPI.

	Coefficient	95% Confidence interval	p-value	Hazard ratio	95% Confidence interval
CRP ≥75.5 mg/L	0.41	-0.45 to 1.26	0.35	1.50	0.64 to 3.52
IPI ≥4	0.58	-0.21 to 1.37	0.15	1.79	0.81 to 3.95
CD4 <150 cells/ul	0.66	-0.20 to 1.52	0.13	1.93	0.82 to 4.56

## 4 Discussion

In this study, we have shown derangements of several immunological proteins in a cohort of patients with DLBCL and a high HIV-prevalence. Levels of the pro-inflammatory cytokine IL-6 and the acute phase protein CRP were elevated in 100% and >90% of the included patients, respectively, and were significantly higher among the people living with HIV. IL-6 functions as a B-cell growth factor, and promotes B-cell differentiation into antibody producing plasma cells (Burger, 2013). IL-6 levels have been shown to be elevated within the tumour micro-environment (TME) of DLBCL, where it is produced by reactive immune and stromal cells (particularly macrophages and endothelial cells) (Emilie et al., 1992), as well as by the tumour cells directly. Haswah et al. demonstrated expression of gp130 [a subunit of the IL-6-receptor (IL-6R)] on the tumour cells of 77/114 (67.5%) patients with DLBCL, with a higher frequency of IL-6R expression on tumours with an activated B-cell COO (Hashwah et al., 2019). Furthermore, they showed that IL-6 promoted engraftment and dissemination of IL-6R expressing tumour cells in a mouse model, as well as promotion of MYC-driven lymphomagenesis (Hashwah et al., 2019). Serum levels of IL-6 are highly correlated with the levels within the TME, suggesting that this is their source (Voorzanger et al., 1996). CRP is made by the liver in response to pro-inflammatory cytokines (such as IL-1beta and IL-6) (Hart et al., 2020). Both IL-6 and CRP levels were very much higher in this study (median 381.6 pg/ml and 93 mg/L) than those reported previously in DLBCL [ranging from a median of 0.23pg/ml to 26.9 pg/ml for IL-6 (Načinović-Duletić et al., 2008; Giachelia et al., 2012; Tisi et al., 2014) and 22.4 to 30.9 mg/L for CRP (Cao et al., 2012; Adams et al., 2015)]. As would be anticipated, we found a positive correlation between IL-6 and CRP levels (Načinović-Duletić et al., 2008), and as has been reported previously, high CRP levels were related to advanced disease stage (Cao et al., 2012; Troppan et al., 2014; Adams et al., 2015), LDH levels (>200 U/L) (Adams et al., 2015), low Haemoglobin levels (Adams et al., 2015) and poorer survival (Cao et al., 2012; Troppan et al., 2014; Adams et al., 2015; Sun et al., 2018; Gradel and Larsen, 2022) (although this was not independent of the CD4 count or IPI). However, in contrast to previous reports, the CRP showed no correlation with the IPI (Cao et al., 2012; Troppan et al., 2014; Gradel and Larsen, 2022), and there was no association between IL-6 levels and the Haemoglobin (Tisi et al., 2014), LDH level, disease stage, β2 microglobulin level (Načinović-Duletić et al., 2008) or the COO. Interestingly, there was a negative correlation between the IL-6 level and bulky disease in this study. Coupled with the lack of association with disease stage and other markers of disease burden, our findings suggest that circulating IL-6 may derive from sources other than the TME in the setting of HIV-DLBCL (possibly as a result of chronic immune stimulation consequent upon recurrent/chronic infection). This is likely also true for the CRP, particularly in light of the inverse correlation between the CRP level and the CD4 count. While high levels of IL-6 may well contribute to DLBCL pathogenesis among people living with HIV, this finding did not show the same association with survival reported elsewhere (Načinović-Duletić et al., 2008; Giachelia et al., 2012). While this could be due to intrinsic differences in the tumour biology in the setting of HIV-DLBCL (due to low IL-6R tumour expression for instance), it more likely reflects the fact that high IL-6 levels are less often tumour-derived in this setting, and are therefore a less clear biomarker of the underlying tumour biology. As such, high IL-6 levels are not predictive of tumour chemosensitivity and behaviour in HIV-DLBCL.

Similarly, levels of the anti-inflammatory cytokine IL-10 were very much higher in this cohort (elevated in all patients, median 184.5 pg/ml) as compared to that described in patients with DLBCL in Europe and the United States of America (USA) (ranging from 4.9-35 pg/ml) (Lech-Maranda et al., 2006; Načinović-Duletić et al., 2008; Gupta et al., 2012), with many patients in studies from other parts of the world having undetectable IL-10 levels. IL-10 is made by monocytes, TH2 helper cells, Tregs, as well as some activated B- and T-cells (Béguelin et al., 2015), functioning in the homeostatic down-regulation of inflammation following a pro-inflammatory stimulus. In addition, elevated IL-10 levels in DLBCL have been shown to derive from upregulated IL-10 gene expression in the tumour cells, particularly in those with an Activated B-cell (ABC) COO (Béguelin et al., 2015; Stirm et al., 2021). This is driven by Nuclear factor kappa-light-chain-enhancer of activated B cells (NFKB) activation (Béguelin et al., 2015), which in turn is often mediated by Myeloid differentiation primary response 88 (MYD88) and CD79A/B mutations (Stirm et al., 2021). In addition, IL-10 signalling is magnified by amplification of subunits of the IL-10-receptor gene in a proportion of cases (Béguelin et al., 2015), and IL-10 synthesis by the tumour cells is further driven as part of an autocrine positive feedback loop (Béguelin et al., 2015). In our study, IL-10 levels were inversely correlated with the haemoglobin and albumin levels, as reported elsewhere (Lech-Maranda et al., 2006), positively correlated with the IL-6 and monocytes counts, with marginally significant associations with disease stage and CRP. However, in contrast to reports from elsewhere, IL-10 levels were not correlated with the IPI, LDH, performance status, age or β2-microglobulin levels (Lech-Maranda et al., 2006; Načinović-Duletić et al., 2008; Gupta et al., 2012), and no association was seen between the IL-10 level and the COO or the circulating Treg count. The weak association between IL-10 levels and markers of disease bulk/COO, together with the association seen between IL-10 levels and the monocyte count and IL-6 level, suggest that serum IL-10 is likely at least partially monocyte derived in our setting (possibly due to infection-related immune activation among the people living with HIV). Although generally immunosuppressive as a result of TH1 and other pro-inflammatory cytokine depression (Béguelin et al., 2015) and the promotion of Treg survival and immunosuppressive activity (Stirm et al., 2021), IL-10 activates Janus kinase (JAK)-signal transducer and activator of transcription (STAT) (JAK-STAT) signalling in B-cells (Gupta et al., 2012; Béguelin et al., 2015; Stirm et al., 2021), promoting their proliferation and survival. High IL-10 levels are also associated with recruitment of Tregs to the TME (Stirm et al., 2021), thus promoting immune evasion by the tumour cells. All of these factors are likely to account for the negative correlation with survival seen in relation to high IL-10 levels in this study, a finding in line with several previous reports (Lech-Maranda et al., 2006; Načinović-Duletić et al., 2008; Gupta et al., 2012; Béguelin et al., 2015; Stirm et al., 2021). The fact that IL-10 was elevated in all tested patients in this cohort may well contribute to the very poor survival rates we observed. Notably, IL-10-receptor blockade has been shown to induce cell death in DLBCL cell lines (Béguelin et al., 2015) and mouse models (Stirm et al., 2021). In fact, several avenues of IL-10 inhibition (including anti-IL-10 monoclonal antibody therapy and small molecule inhibitors of both JAK2 and NFKB) are being explored as a novel therapeutic approach in DLBCL (Béguelin et al., 2015). Given the findings in our study, further assessment of these agents in the setting of HIV-DLBCL would be of interest. Interestingly, IL-10 is thought to increase ferritin translation in activated monocytes (Tilg et al., 2002), as was borne out by the significant correlation between IL-10 and ferritin levels in this cohort. Ferritin has an anti-oxidant effect in the TME, protecting the tumour cells from the generation of reactive oxygen species induced by free iron (Alkhateeb and Connor, 2013). It is thought that this anti-oxidant capacity may cause chemoresistance in patients with high ferritin levels, as the cytotoxic effects of several chemotherapeutic agents is known to be partially attributable to the induction of oxidative stress (Alkhateeb and Connor, 2013). As such, high ferritin levels are well documented to be associated with inferior survival in DLBCL (Ghesquieres et al., 2010; Kim et al., 2020; Shen et al., 2021), as was the case in this study.

Unlike IL-6 and IL-10, the serum levels of TGFβ did not differ statistically according to HIV-status, but high levels were significantly associated with poorer outcomes. The functions of TGFβ are pleitropic, including regulation of tissue growth and of the immune system (Kubiczkova et al., 2012). It is a potent negative regulator of haemopoiesis, and generally has anti-proliferative effects (Kubiczkova et al., 2012), mediated partially through down-regulation of the MYC gene (Warner et al., 1999). It also plays a role in B-cell differentiation, is a negative regulator of NFKB signalling and is produced by Tregs (Kubiczkova et al., 2012), neutrophils (Grotendorst et al., 1989) and monocytes (Grotendorst et al., 1989). TGFβ signalling is thought to have both pro- and antiapoptotic activity in B-cells, depending on their location and stage of development (Timmins and Ringshausen, 2022). In contrast to our findings, previous studies in the setting of DLBCL have shown higher TGFβ levels to have a positive impact on tumour control. For instance, TME expression profiling studies have shown recurring expression signatures characterised by increased TGFβ signalling which are associated with superior outcomes (The “Stromal 1” (Haro and Orsulic, 2018) and “Mesenchymal” (Kotlov and Bagaev, 2021) signatures). However, aberrations rendering tumour cells intrinsically resistant to TGFβ are reportedly common in B-cell lymphomas, either due to reduced expression of the TGFβ receptor (particularly among EBV-positive neoplasms) (Inman and Allday, 2000), or aberrant downregulation of its downstream targets (Stelling et al., 2018). Furthermore, in the setting of Follicular lymphoma, TGFβ secretion by both tumour cells and T-cells within the surrounding TME have been reported to cause Treg expansion (Yang et al., 2013), suppression of helper T-cell generation (Yang et al., 2013) and induction of T-cell exhaustion (Yang et al., 2014), which is associated with poorer survival outcomes (Yang et al., 2014). In addition, TGFβ derived from lymphoblasts in B-cell lymphoblastic leukaemia has been shown to cause natural killer cell dysfunction (Rouce et al., 2016), thus facilitating immune evasion by the tumour cells. Coupled with possible innate resistance to TGFβ in the tumour cells, this immune-evasive potential may account for the negative impact associated with high TGFB levels seen in this study. This hypothesis is supported by the significant relationship seen between TGFβ levels and peripheral blood numbers of Helios and CD39-expressing Tregs (markers of more potent immunosuppressive Treg potential), as well as the marginally significant correlation between TGFβ and Kynurenine levels (a metabolite which is cytotoxic to T-cells, and has an immune tolerising effect) (Suchard et al., 2020). Notably, TGFβ levels also showed a significant correlation with the neutrophil count, which may account for the poorer survival we showed previously in this cohort among patients with a neutrophilia (Vaughan et al., 2023). Interestingly, TGFβ resistance has been shown to be linked to epigenetic silencing of the expression of the TGFβ receptor, which can be partially reversed with demethylating agent therapy (thus resensitising tumour cells to the pro-apoptotic effect of TGFβ) (Chen et al., 2007). Since HIV-DLBCL is often EBV-associated [which has been shown to be linked to low expression of the TGFβ receptor in other B-cell lymphomas (Inman and Allday, 2000)], further investigation as regards the frequency of epigenetic silencing of TGFβ-related genes and the potential value of demethylating agent therapy in the setting of HIV-DLBCL would be of interest.

While both SFLC and Kynurenine levels were considerably elevated in this study, neither showed the significant association described between these variables and survival in the international literature (Yoshikawa et al., 2010; Maurer et al., 2011; Jardin et al., 2013; Han et al., 2014; Chen et al., 2020). There was elevation of one/both free light chains in 76.6% of the patients in this cohort, the majority of whom had elevation of both Kappa and Lambda light chains (specifically among the people living with HIV). The frequency of elevation of both light chains (60.9%) was considerably higher than that reported previously among patients with DLBCL in China (23.2%) (Han et al., 2014) and France (4.2%) (Jardin et al., 2013), while the frequency of an abnormal SFLC ratio was similar to previous reports [14.1% versus 4.6% in China (Han et al., 2014), 9.3% in France (Maurer et al., 2011; Jardin et al., 2013) and 14% in the USA (Maurer et al., 2011)]. As described previously among patients with auto-immune pathology, FLC levels showed significant correlations with other markers of B-cell activation (β2-microglobulin and IgG levels) (Gottenberg et al., 2007), and were also significantly inversely correlated with the CD4 count. These findings likely reflect the effects of chronic antigenic stimulation in people with advanced HIV-infection. IDO is activated by pro-inflammatory cytokines, and catalyses conversion of the amino acid tryptophan to kynurenine (François et al., 2012). The resultant tryptophan depletion causes pro-inflammatory T-cell apoptosis, as well as induction of resting T-cell differentiation into regulatory Tregs. IDO activity (reflected by the KTR) is known to be elevated in people living with HIV, with levels reportedly showing a correlation with HIV stage (Adu-Gyamfi et al., 2019). While we found significantly higher KTR and Kynurenine levels in the people living with HIV, there was no significant association with the CD4 count or any other HIV-related variables. As with the CRP and IL-6 levels, SFLC is likely increased predominantly due to the effects of chronic immune stimulation in the setting of HIV-DLBCL, while increased IDO activity is a probable product of the homeostatic activation of negative feedback pathways to dampen this perpetual immune activity.

As we have reported previously (Vaughan et al., 2022; Vaughan et al., 2023), survival rates in this cohort were poor as compared to those reported in high income countries (5 year survival rates over 60%) (Sehn and Salles, 2021), as well as in two regional studies, one performed in Malawi among 86 adult patients with a HIV-seropositivity rate of 59% (2 year survival rate of 38%) (Painschab et al., 2019), and the other in a South African study among 36 patients with HIV-DLBCL (2 year overall survival ~40%) (de Witt et al., 2013). This was at least partially attributable to a very high early death rate (~30%), with over half of the patients who demised early doing so before they could be given chemotherapy. Such patients often do not meet the inclusion criteria for clinical trials or demise before they can be enrolled, and may consequently be under-represented in the published literature. Since the patients who died early had significantly higher LDH levels with lower CD4 counts and less frequent ART exposure, this may represent a biologically distinct, more aggressive form of DLBCL associated with advanced-stage HIV infection. Further study of the tumour molecular landscape of this subgroup would be of interest. A possible additional contributor to the suboptimal survival seen here is the very limited use of rituximab among the people living with HIV in our centre owing to resource constraints. Furthermore, the COVID-19 pandemic had a considerable impact on the South African health care system over the course of this study, likely further compromising optimal care.

Limitations of this study include the relatively small sample size (particularly the number of patients in whom cytokine testing and Treg enumeration was performed), the general late presentation of all included patients (irrespective of HIV-status), and the suboptimal therapy available in our setting. Cautious interpretation within the bounds of these limitations is required.

## 5 Conclusion

Derangements of immunological proteins are very common in the setting of HIV-DLBCL, and have a differential impact on survival as compared to that reported in the international literature. CRP, IL-6, SFLC levels and IDO activity are not independently associated with survival in this setting, likely because these are predominantly a product of chronic immune stimulation due to longstanding/recurrent infections in people living with HIV. In contrast, TGFβ and IL-10 were significantly associated with survival in this cohort independently from the IPI and CD4count; further exploration of the directed targeting of these cytokines in HIV-DLBCL would be of interest.

## Data availability statement

The raw data supporting the conclusions of this article will be made available by the authors, without undue reservation.

## Ethics statement

The studies involving humans were approved by Human Research Ethics Committee of the University of theWitwatersrand. The studies were conducted in accordance withthe local legislation and institutional requirements. Informed consent was obtained from all participants from whom peripheral blood was collected solely for the purposes of the study, as well as from all patients on whom testing was performed on residual samples, provided they had not demised in the interim.

## Author contributions

JV: Conceptualization, Data curation, Formal analysis, Funding acquisition, Investigation, Methodology, Writing – original draft. MP: Supervision, Writing – review & editing. MS: Methodology, Resources, Writing – review & editing. MG: Investigation, Methodology, Writing – review & editing. HR: Investigation, Methodology, Writing – review & editing. WH: Investigation, Methodology, Writing – review & editing. TS: Investigation, Methodology, Writing – review & editing. TW: Supervision, Writing – review & editing.
