# Supplementary material for: Derangements of immunological proteins in HIV-associated diffuse large B-cell lymphoma: the frequency and prognostic impact
Source: Front Cell Infect Microbiol. 2024 Apr 3;14:1340096. doi: 10.3389/fcimb.2024.1340096 (PMC11021765; doi:10.3389/fcimb.2024.1340096)
Supplement: Supplementary file 1 [file DataSheet_1.docx]

*Supplementary Material*

# Supplementary Data

This is a substudy of a project entitled “Evaluation of the association between tumour enrichment with M2-macrophages and survival among South African patients with Diffuse Large B-cell Lymphoma”, an inclusion criterion for which included the presence of an adequate diagnostic biopsy sample for further immunohistochemical work-up. Further blood testing was not possible in a number of patients included in this study for several reasons (summarized in Supplementary Figure 1):

- Owing to resource constraints, IL6, IL10 and TGFβ testing was possible in only 36 patients. This was prioritized in patients who had either not been commenced on corticosteroids/chemotherapy, or in those who had been initiated on corticosteroid prior to their biopsy (10 patients). The cytokine levels, median survival times and survival rates were confirmed to be similar between those on corticosteroids as compared to those who were not. In addition, there was no significant difference in the median survival time or the survival rate in those in whom cytokine levels were measured as compared to those in whom this was omitted.
- Access to inpatients was restricted during part of this study due to COVID-19 related measures, so that blood could not be collected specifically for research purposes. As such, the testing was dependent on blood samples submitted for routine diagnostic tests.
- Some of the testing was unsuccessful due to random technical errors.
- Many of the routine tests assessed (such as ferritin, CRP, LDH, B2-microglobulin, CD4, HIVVL, etc) were not requested by the attending clinicians.

SFLC testing unsuccessful due to lipaemia in 1 patient

**Supplementary Figure 1:** Depiction of sample inclusion and exclusion

Excluded

Appropriate tube unavailable for SFLC and IDO testing in 12 and 15 patients, respectively

Approximately 24 patients did not have an adequate biopsy for inclusion in the parent study

76 patients had an adequate biopsy

IL6, IL10 and TGFB levels measured in only 36 patients due to resource constraints.

Ferritin and CRP levels were not requested in 15 and 9 patients, respectively.

Approximately 100 patients were referred to the CHBAH with DLBCL over the study period
